# Strategic use of telemedicine for restarting urological outpatient services during COVID-19 pandemic

**DOI:** 10.1186/s12301-020-00091-0

**Published:** 2020-11-04

**Authors:** Devanshu Bansal, Samit Chaturvedi, Anant Kumar

**Affiliations:** Department of Urology, Renal Transplantation, Robotics and Uro-oncology, Max Hospital, Saket, New Delhi, 110017 India

India reported its first case of coronavirus disease (COVID-19) on 30th January 2020. The Indian Government declared nationwide lockdown on 25th March 2020, which continued till 31st May 2020 [1]. Thereafter, most of the country was opened with some relaxations except in red zones and containment areas. The lockdown has been essential in building government capacity to test and treat COVID-19 patients and manufacture personal protective equipment (PPE). There have also been public awareness campaigns regarding social distancing, face mask use, hand and respiratory hygiene and judicious use of public places. After the initial panic, the healthcare workers have now equipped themselves with knowledge about diagnosing and treating COVID-19 and proper use of PPE. However, it is clear that the lockdown is not a long-term solution and prolonged curbs to healthcare can have long lasting adverse impact on routine patient care. Amidst the pandemic, routine urologic services cannot be persistently postponed. Outpatient services form the backbone of restarting urologic services as the lockdown is eased and specific measures are required to ensure provision of quality services while ensuring safety of both patients and healthcare workers.

In various countries, there has been rapid development and adoption of telemedicine practices during current time [2]. In the pre-pandemic era, telemedicine has been helpful in providing expert advice without the hassle of travelling. It has offered a possibility of getting specialist opinion in remote and rural areas and for older patients who have limitations for travel [3]. However, its use had not been widespread due to inherent challenges of acquiring new technology and unclear reimbursement rules by the insurance providers [3]. During the lockdown, the above advantages with the addition of provision of maintaining the doctor–patient contact without the risk of transmission of COVID-19 have led to many healthcare providers incorporating the facility in their routine practice [2]. Studies have shown that telemedicine proves satisfactory for both doctors and patients in a majority proportion with its efficacy non-inferior to face-to-face visits [2, 4]. Telemedicine can lead to significant cost savings compared to in-person consultation, mainly in the form of transportation cost, patient time and staff cost [5]. The Ministry of Health and Family Welfare has given detailed guidelines regarding practicing telemedicine in India [6]. Salient points include proper identification of the registered medical practitioner and the patient, selection of appropriate mode for communication pertaining the clinical context (video consult for first visit, audio/text consult may be used for follow up visits), patient consent (implied if patient initiates the tele-consult), maintenance of proper audio-visual channel for adequate patient evaluation, formulation of a provisional diagnosis, maintaining adequate code of conduct, professional advice (should be the same as would be given in a face-to-face consult) and record keeping for future assessments. It is also of utmost importance to ensure privacy and safety of patient identity and medical information from hacking and unintentional exposure.

Telemedicine requires some initial investment by the healthcare providers, patient education and cooperation and support by information technology department. Although audio-visual platforms such as WhatsApp, Skype and FaceTime are allowed for use under the national guidance, it will be best to use dedicated telemedicine software [7] to ensure that adequate healthcare standards are met [2]. Special challenges may be encountered in India, including a majority rural population, which might not be well versed with the telemedicine platform, walk-in patients and lack of reimbursement of tele-services by insurance companies.

While telemedicine is a powerful tool, in a country like India, in-person outpatient services are irreplaceable to restart urological care and should get adequate focus during exit from lockdown, while ensuring social distancing and good hygiene practices.

## Recommendations for outpatient services

**Fig. 1:**
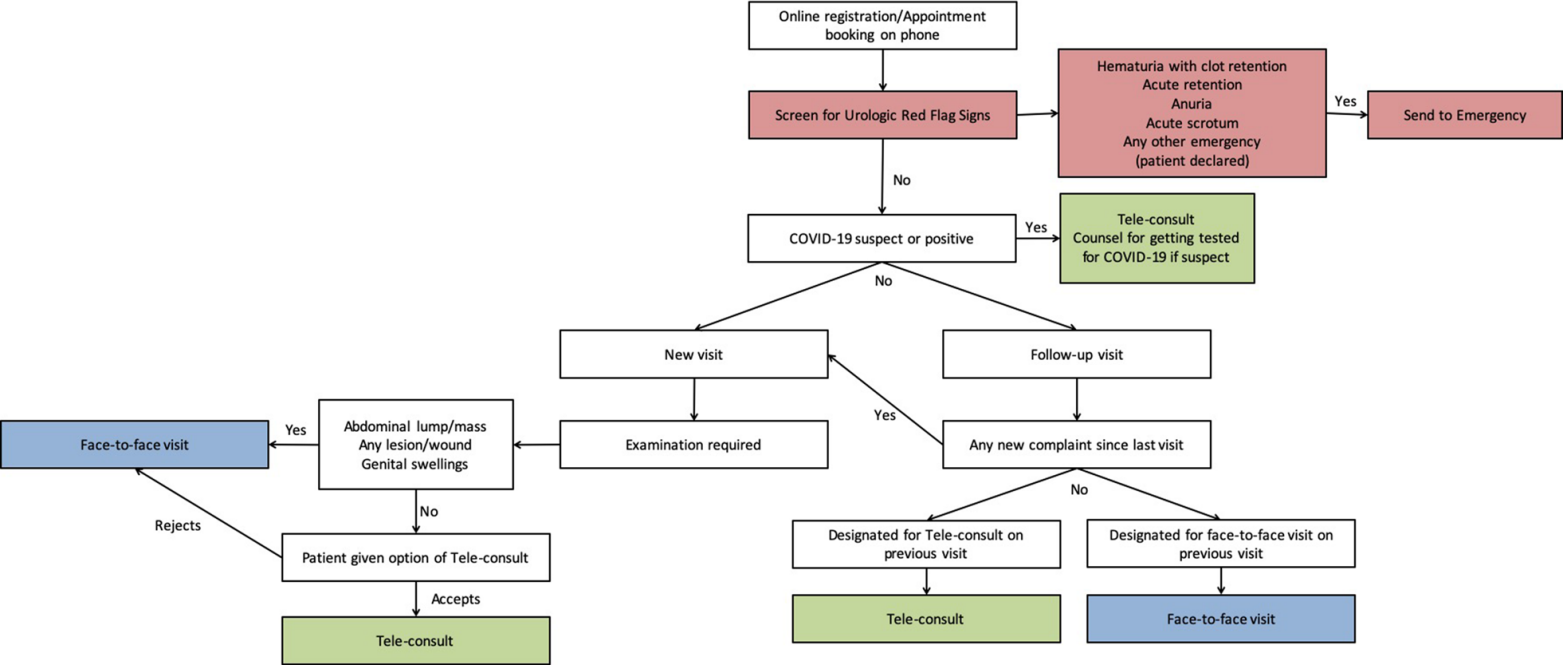
Proposed algorithm for incorporation of telemedicine in urologic outpatient care

**Fig. 2:**
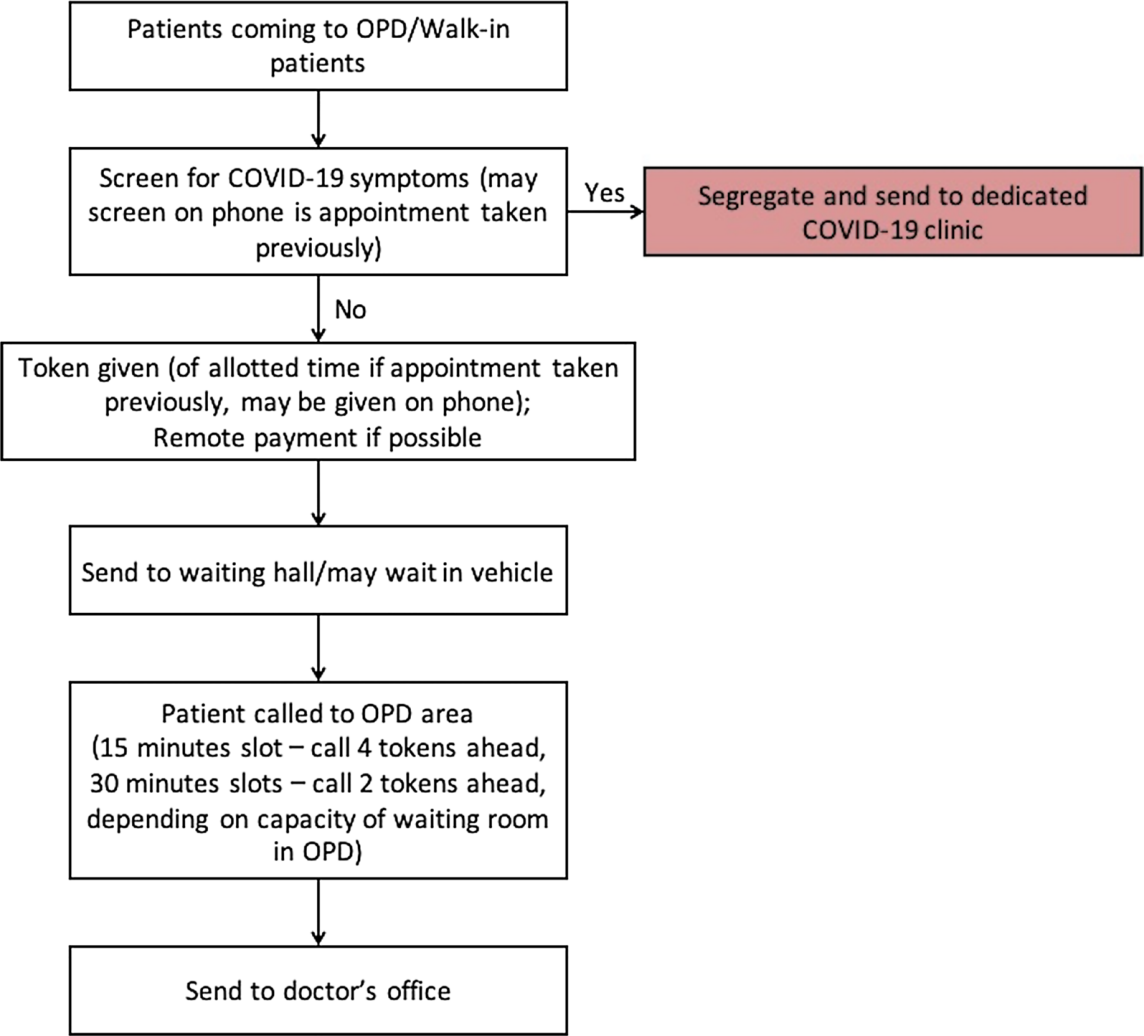
Proposed algorithm for management of in-person outpatient clinic visits

We believe that telemedicine may be here to stay and it should be practiced wherever possible as either a replacement or adjunct to face-to-face patient visits. We provide a novel flowchart for incorporating telemedicine into urologic practice in Fig. 1. On the other hand, it is inevitable that many patients may visit the hospital without a prior appointment (walk-in patients). Therefore, full precautions need to be taken, including social distancing, maintaining hand hygiene, limiting the waiting time before the doctor visit and electronic payment wherever possible. We propose a model for provision of outpatient services to both walk-in patients and those booked for a face-to-face appointment in Fig. 2. Appointments should be properly scheduled to avoid excessive waiting time. Recommended PPE should be used by doctors in their outpatient clinic as suggested by the government of India (including masks and gloves) [8]. Low-cost adjunctive protective solutions, such as use of plastic or glass screens, may be placed between the doctor and the patients so as to limit any direct aerosol spread. Washable gowns or hospital scrubs may be worn by doctors in the outpatient clinic to limit droplet contamination of their personal clothes. Gloves must be worn whenever there is a need to examine a patient, and these should be discarded immediately after. In the case of oral/anal cavity examination, face-shields should be worn in addition to N95 masks. Hand hygiene should be practiced between each patient visit, and frequent sanitization of the clinic should be done. Mask wearing and hand sanitization should be mandatory for patients. Patients not requiring any assistance should be encouraged to visit the doctor’s clinic without any attendant. In case the attendant needs to discuss something with the doctors, the patient may be sent out and then attendant entertained, to limit exposure. Social distancing measures to be reinforced at the start of the visit in order to avoid exposure in case of an enthusiastic patient who may get up from the chair to show his documents. It is also suggested that the reports may be browsed before the patient enter the doctor’s chamber so as to limit the person-to-person contact time. To conclude, outpatient urological care may be restarted using a strategic reopening plan and adequate precautions.

## Data Availability

Not applicable.

## Abbreviations

COVID-19: Coronavirus disease
PPE: Personal protective equipment
